# The effectiveness of vaginal progesterone in reducing preterm birth in high-risk patients diagnosed with short cervical length after 24 weeks: A retrospective cohort study

**DOI:** 10.3389/fmed.2023.1130942

**Published:** 2023-03-02

**Authors:** Danielle Luxenbourg, Shay Porat, Roberto Romero, Dror Raif Nesher, Rani Haj Yahya, Yishai Sompolinsky, Hila Hochler, Yossef Ezra, Doron Kabiri

**Affiliations:** ^1^Department of Obstetrics and Gynecology, Hadassah Medical Organization and Faculty of Medicine, Hebrew University of Jerusalem, Jerusalem, Israel; ^2^Perinatology Research Branch, Division of Obstetrics and Maternal-Fetal Medicine, Division of Intramural Research, Eunice Kennedy Shriver National Institute of Child Health and Human Development, National Institutes of Health, US Department of Health and Human Services, Bethesda, MD, United States; ^3^Department of Obstetrics and Gynecology, University of Michigan, Ann Arbor, MI, United States; ^4^Department of Epidemiology and Biostatistics, Michigan State University, East Lansing, MI, United States; ^5^Center for Molecular Medicine and Genetics, Wayne State University, Detroit, MI, United States; ^6^Detroit Medical Center, Detroit, MI, United States

**Keywords:** admission to neonatal intensive care unit, prematurity, preterm delivery, preterm labor, progesterone, progestogen, short cervix

## Abstract

**Objective:**

To assess the impact of progesterone treatment on maternal and neonatal outcomes in women with a history of preterm birth and short cervical length diagnosed after 24 weeks of gestation.

**Methods:**

A retrospective cohort study included women with a history of preterm birth and a transvaginal sonographic cervical length measurement of ≤ 25 mm, diagnosed between 24^+0^ and 33^+6^ weeks of gestation. Exclusion criteria included prior progesterone treatment, cervical cerclage, or pessary. The study population was divided into the progesterone treatment group and the non-treatment group.

**Results:**

The study included 104 women, with 46.2% (48/104) receiving progesterone treatment and 53.8% (56/104) not receiving treatment. The rate of spontaneous preterm birth before 37 weeks of gestation was 43% (24/56) in the non-treatment group and 31% (15/48) in the progesterone treatment group (*P* = 0.14); the rate of spontaneous preterm birth before 34 weeks was 7% (4/56) in the non-treatment group and 0% (0/48) in the progesterone treatment group (*P* = 0.05). Progesterone treatment was associated with a significant decrease in neonatal intensive care unit admissions (OR 0.20, 95% CI 0.05–0.74) and in the neonatal hospitalization period (mean difference in days 2.43, 95% CI 0.44–4.42). The risk of recurrent spontaneous preterm birth was highest (71%) among women with two or more previous preterm deliveries who did not receive progesterone treatment, and lowest (24%) among women with one previous preterm delivery who received progesterone treatment.

**Conclusion:**

Progesterone treatment was associated with a reduction in rates of spontaneous preterm birth before 34 weeks of gestation, neonatal intensive care unit admission, and neonatal length of stay in high-risk patients, even when initiated after 24 weeks of gestation.

## Synopsis

The administration of progesterone to high-risk women is associated with lower rates of spontaneous preterm birth and NICU admissions.

## Introduction

Preterm birth is the leading cause of perinatal mortality and morbidity (1, 2). Women with a history of spontaneous preterm birth (sPTB) have a recurrence in 21%−57% of subsequent pregnancies and are considered a high-risk population (1, 3–5). To decrease the rate of sPTB in these patients, preventive treatment with progestogens has been proven to be effective (6–10).

Cervical length measurement between 18 and 24 weeks of gestation is the most powerful predictive tool for sPTB (3, 5, 6, 11), and its utility for sPTB prevention has been repeatedly demonstrated in patients with a short cervix treated with vaginal micronized progesterone. This treatment reduces the risk of sPTB by 31%−49% (10, 12–14). Several studies demonstrated that short cervical length measured after 24 weeks of gestation is a risk factor for sPTB (15–18); however, the effect of vaginal progesterone treatment to prevent sPTB in this subset of pregnant women has not been examined in any prospective or retrospective study (18).

Some patients with prior preterm delivery do not receive any preventative treatment despite its wide recommendation, due to physician's preference, patient's preferences, or the patient's inability to comply. These patients may pose a management dilemma when referred for treatment after 24 weeks of gestation with the new diagnosis of a sonographic short cervical length.

The aim of this study was to assess the effect of vaginal progesterone treatment on the prevention of sPTB and adverse neonatal outcomes in women with previous preterm birth and a short cervix newly diagnosed after 24 weeks of gestation.

## Methods

This retrospective cohort study was conducted in the maternal-fetal units of two university teaching referral hospitals with approximately 14,000 deliveries per year. The study population consisted of women with a singleton pregnancy and previous preterm birth, diagnosed with a cervical length ≤ 25 mm from 24^+0^ to 33^+6^ weeks of gestation between 2010 and 2017 (19). Maternal and neonatal parameters were compared between women with vaginal progesterone treatment (i.e., progesterone treatment group) and women with no progesterone treatment (i.e., non-treatment group). This study was approved by the Institutional Review Board (0446-20-HMO). As a retrospective study based on existing data and containing no identifying details, no informed consent was required.

Cervical length was quantitatively assessed by trained sonographers with a minimum of 10 years of experience, utilizing transvaginal ultrasound imaging in patients who were in the dorsal lithotomy position and had an empty bladder. The vaginal probe was positioned in the anterior fornix, and the internal and external OS, as well as the entire cervical canal, were visualized. Calipers were then placed between the ora to obtain the shortest and most accurate measurement, which was recorded. The ultrasound equipment utilized included the Voluson 730 Expert, E6, E8, and E10 (manufactured by GE Healthcare in Zipf, Austria) and the Samsung Accuvix A30 (manufactured by Samsung Co., Ltd. in Seoul, South Korea), both of which were equipped with vaginal probes.

Women were excluded from this study if they were diagnosed with a short cervical length measurement prior to 24^+0^ weeks of gestation, had a progestogen prescription prior to 24^+0^ weeks, underwent pessary or cervical cerclage placement during the current pregnancy, and had either a medically indicated preterm delivery or delivered on the same day of the short-cervix diagnosis.

Demographics and obstetrical history were obtained from the electronic medical records of the patients. The primary outcome measured was the rate of sPTB, defined as the spontaneous onset of labor resulting in birth before 37 weeks of gestation. Secondary outcomes consisted of sPTB by <34, <32, and <28 weeks of gestation; mean gestational age at diagnosis and delivery; lag from short cervical length diagnosis to delivery; and mode of delivery. Neonatal outcomes included the duration of the neonatal hospitalization period; Apgar score <7 at 5 min; birth weight; neonatal intensive care unit (NICU) admission; respiratory distress syndrome; intra-ventricular hemorrhage; necrotizing enterocolitis; retinopathy of prematurity; bronchopulmonary dysplasia; and neonatal mortality. The modes of delivery were categorized into vaginal delivery, operative delivery, or cesarean delivery. Neonatal hospitalization was measured in days from birth to discharge. The Apgar score after 5 mins was also reported in the study. Admission to the NICU was registered for any period during the primary period after birth. Respiratory distress syndrome was defined by cyanosis, tachypnea, and respiratory failure shown in blood gas and chest radiography (20). Intra-ventricular hemorrhage was diagnosed and graded according to the Papile et al. (21) classification. Necrotizing enterocolitis was diagnosed and staged based on Bell's criteria (22) for stages 2 and 3, using clinical and radiographic findings. Retinopathy of prematurity was diagnosed according to the International Classification of Retinopathy of Prematurity (ICROP) (23). Bronchopulmonary dysplasia was diagnosed in infants who were treated with oxygen >21% for at least 28 days based on the need for oxygen supplementation and the gestational age (24). Neonatal mortality was defined as neonatal demise during the first 28 days after delivery.

We performed a sub-analysis according to the time of previous preterm birth (occurrences of previous early preterm birth before 34^+0^ weeks and late preterm birth between 34^+0^ and 36^+6^ weeks of gestation) and the number of previous preterm births (single vs. multiple).

A Chi-square test or Fisher's exact test was used to test the association between two categorical variables. A two-sample t-test or the non-parametric Mann-Whitney test was used to compare quantitative variables between two independent groups. The non-parametric test was applied for non-normally distributed data. The Kaplan-Meier survival model, with the log-rank test for the comparison of survival curves, was applied to assess the effect of categorical variables in the event of premature birth, taking into account the time from cervical length measurement to delivery. The Cox regression model was used to assess the effect of quantitative variables on the event of premature birth, taking into account the time from cervical length measurement to delivery. The Cox regression model was also used as the multivariate model for simultaneously assessing the effect of several variables on premature birth, taking into account the time from cervical length measurement to delivery. All applied statistical tests were two-tailed, and a *P*-value of 5% or less was considered statistically significant. The data were analyzed using Software Package for Statistics and Simulation (IBM SPSS version 24, IBM Corp, Armonk, NY).

## Results

**Figure 1:**
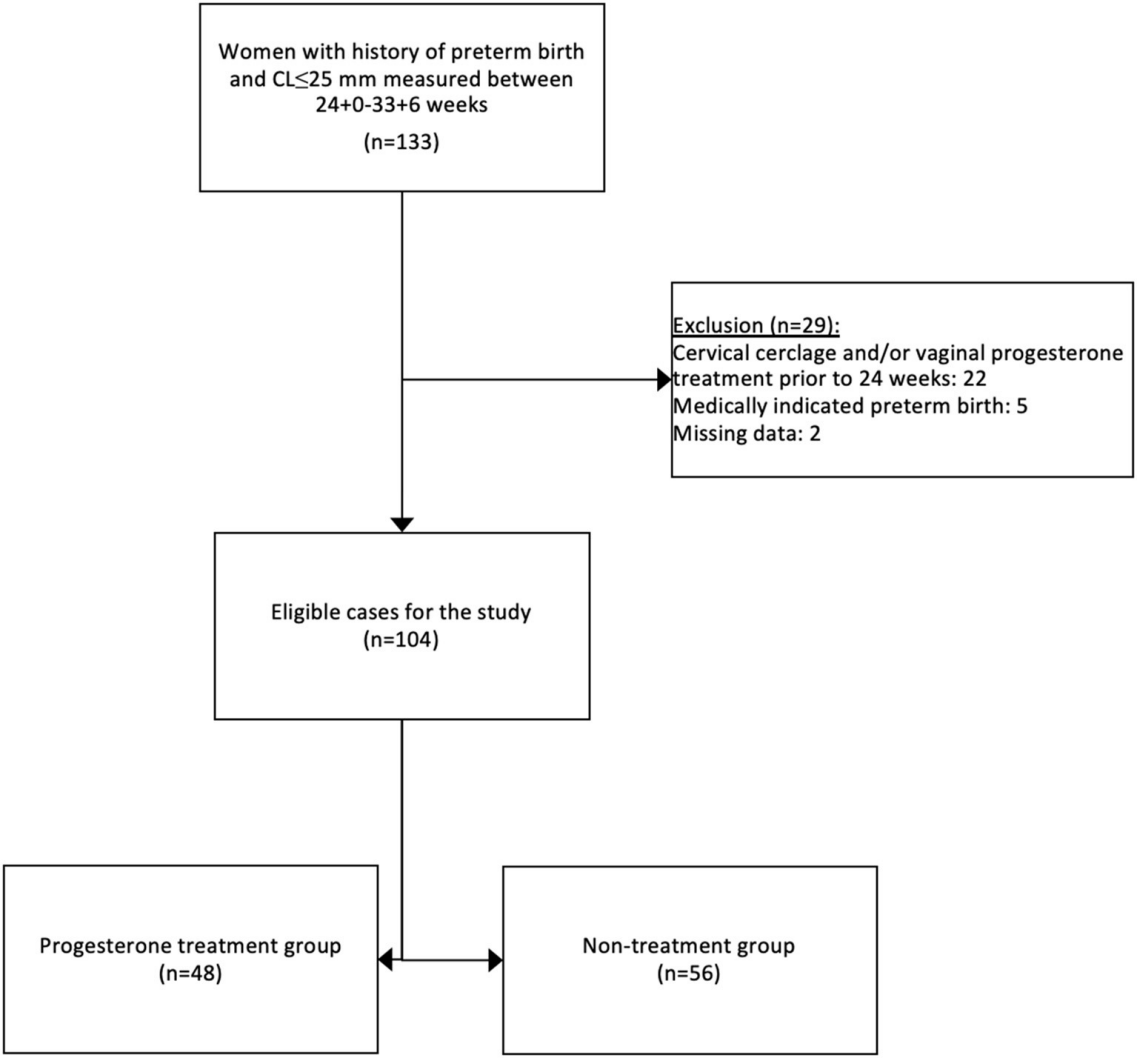
Study design.

One-hundred and thirty-three women with a singleton pregnancy, a history of preterm birth, and a cervical length ≤ 25 mm measured between 24^+0^ and 33^+6^ weeks of gestation were identified through their medical records for this study. After exclusion criteria, 104 cases were eligible for the study cohort as presented in Figure 1. Forty-eight women (46.2%) received vaginal progesterone treatment and composed the progesterone treatment group. Fifty-six women (53.8%) were included in the non-treatment group. Baseline characteristics are listed in Table 1. Demographic and obstetric characteristics were similar between the groups.

**Table 1:** Baseline characteristics of the study population.

**Characteristic**	**Treatment group (*n* = 48)**	**Non-treatment group (*n* = 56)**	***P* value**
Maternal age [years, mean ± SD, (range)]	30.5 ± 5.5	31.6 ± 5.7	0.339
	(19.4–42.6)	(22.5–49.1)	
Parity (median and interquartile range)	2, 2	2, 2	0.683
Previous cesarean delivery [*n* (%)]			0.749
• None	37 (77.1%)	42 (75%)	
• One	8 (16.7%)	8 (14.3%)	
• Two or more	3 (6.2%)	6 (10.7%)	
**Previous preterm birth**			
Number of previous preterm births [*n* (%)]			0.634
• One	34 (71%)	42 (75%)	
• Two or more	14 (29%)	14 (25%)	
° Two	10	10	
° Three	3	2	
° Four	0	1	
° Six	1	1	
Timing of last previous preterm birth			0.72
• Very early preterm (24^+0^-27^+6^)	5 (10%)	4 (7%)	
• Early preterm (28^+0^-33^+6^)	12 (25%)	12 (21%)	
• Late preterm (34^+0^-36^+6^)	31 (65%)	40 (71%)	
Cerclage in previous preterm birth	0	1	–
Preterm birth in precedent birth [*n* (%)]	39 (81%)	43 (77%)	0.578
**Current pregnancy**			
Gestational age at cervical length measurement (weeks, mean ± SD)	29.9 ± 2.7	30.6 ± 2.5	0.201
Cervical length measurement [*n* (%)]			0.105
• Length ≤ 15mm	13 (27%)	8 (14%)	
• Length > 15mm	35 (73%)	48 (86%)	

**Figure 2:**
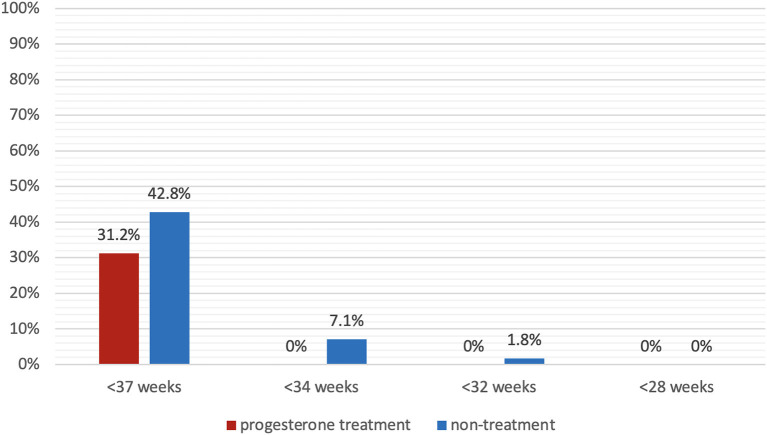
Spontaneous preterm birth rate. Spontaneous preterm birth prior to 37 weeks, 34 weeks, 32 weeks, and 28 weeks of gestation in patients with progesterone treatment (red) and without progesterone treatment (blue).

**Figure 3:**
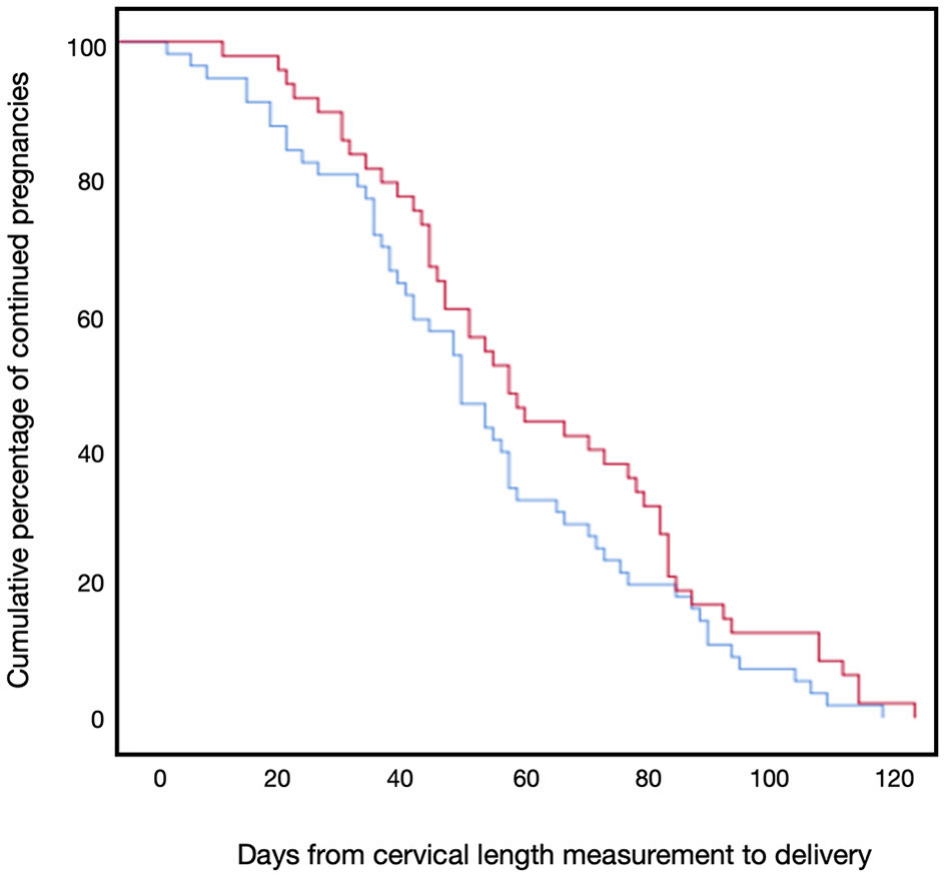
Kaplan-Meier plot for days to delivery. The Kaplan-Meier plot of delivery by days from cervical length measurement of the progesterone treatment group (red line) and the non-treatment group (blue line).

Figure 2 presents the primary outcome, sPTB prior to 37 weeks of gestation, which occurred in 31.2% (15/48) of the progesterone treatment group and 42.8% (24/56) of the non-treatment group (*P* = 0.14). No events of sPTB before 34 weeks occurred in the progesterone treatment group, whereas 7.1% (4/56) of the non-treatment group delivered before 34 weeks of gestation (*P* < 0.05). Figure 3 shows the Kaplan-Meier plot, which displays the lag period from cervical length measurement to delivery among the treatment group juxtaposed with the non-treatment group. The unadjusted hazard ratio for sPTB < 37 weeks of gestation was 0.61 [95% confidence interval (CI) 0.27–1.36] for the treatment group. Maternal outcomes are shown in Table 2.

**Table 2:** Maternal outcomes by progesterone treatment.

	**Treatment group (*n* = 48)**	**Non-treatment group (*n* = 56)**	***P* value**
Gestational age at birth [weeks, mean ± SD, (range)]	37.5 ± 1.6 (34.3–41.6)	37.1 ± 2.2 (31.6–41.7)	0.35
Time from cervical length measurement to delivery [weeks, mean ± SD, (range)]	7.6 ± 3.1 (1.9–14.3)	6.5 ± 3.1 (0.8–13.7)	0.09
Spontaneous preterm birth [*n* (%)]			
• <37 weeks	15 (31.2%)	24 (42.8%)	0.14
• <34 weeks	0	4 (7.1%)	0.05
• <32 weeks	0	1 (1.8%)	1.00
Recurrent preterm birth with			0.35
• One previous preterm birth	8/34 (24%)	14/42 (33%)	
• Two previous preterm births	5/10 (50%)	7/10 (70%)	
• Three and more previous preterm births	2/4 (50%)	3/3 (100%)	
Antepartum corticosteroid administration [*n* (%)]	43 (90%)	48 (86%)	0.55
Mode of delivery [*n* (%)]			0.19
• Spontaneous vaginal delivery	40 (83%)	38 (68%)	
• Operative vaginal delivery	1 (2%)	4 (7%)	
• Cesarean delivery	7 (14%)	14 (25%)	

The Cox proportional logistic regression model for delivery before 37 weeks of gestation included five confounders: progesterone treatment; cervical length ≤ 15 mm; maternal age; the number of previous preterm deliveries; and occurrence of preterm delivery in the antecedent delivery. The adjusted hazard ratio (aHR) of progesterone treatment was 1.539 (95% CI 0.79–3.02, *P* = 0.21), and the aHR for the number of previous preterm deliveries was 2.01 (95% CI 1.04–3.89, *P* = 0.03).

**Figure 4:**
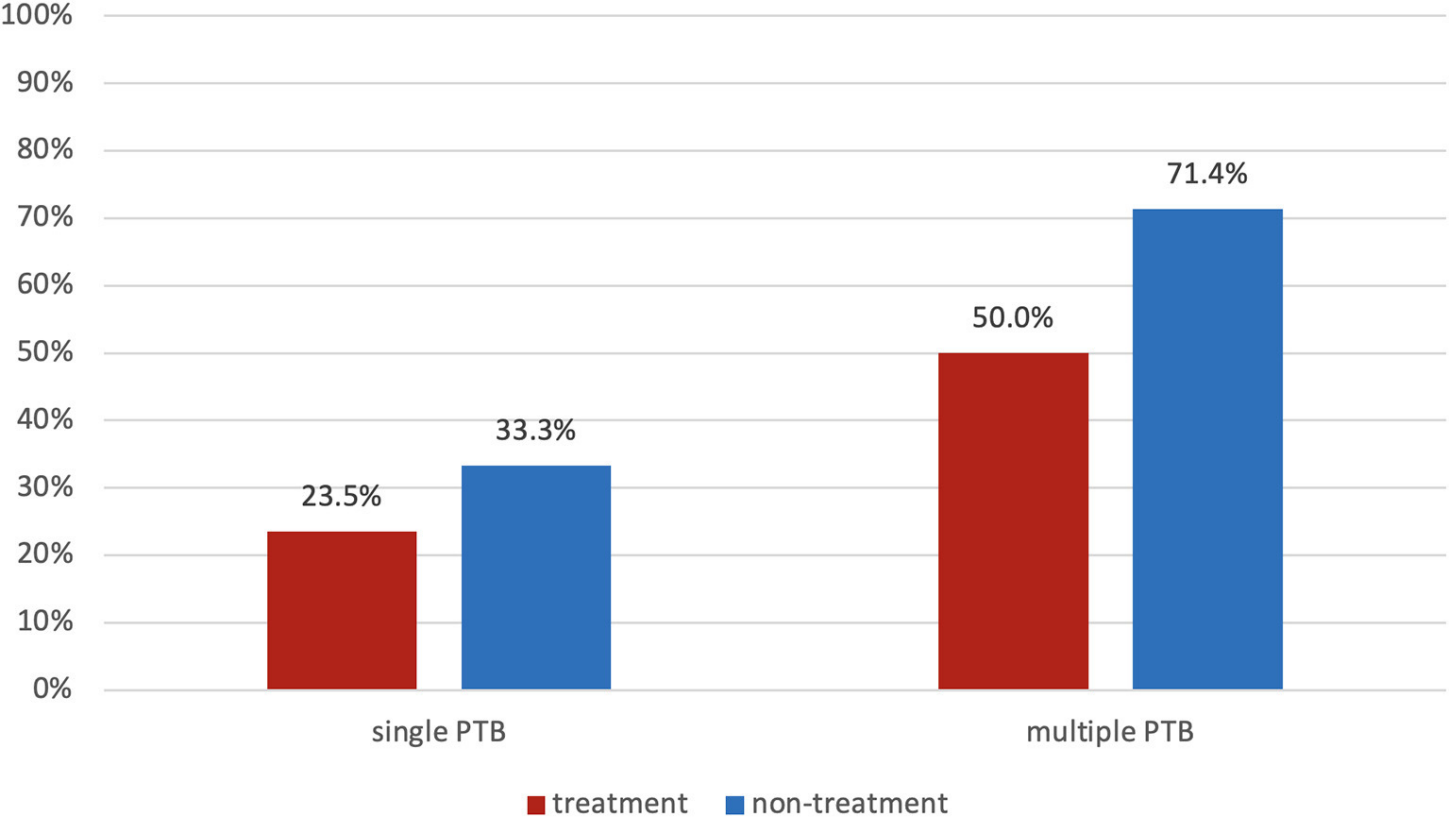
Spontaneous preterm birth rate of patients with a single event or multiple events of prior preterm birth (red—progesterone treatment group; blue—non-treatment group).

A comparison of spontaneous preterm birth rates in high-risk pregnancies with prior preterm births receiving progesterone treatment and non-treatment group is presented in Figure 4. Twenty-eight women had a history of two or more preterm births, and 76 women had a single prior preterm birth. Women with two or more prior preterm deliveries had a higher overall risk for sPTB than women with one prior preterm delivery (aHR 2.1). The sPTB rate was lowest (23.5%) among patients with a single prior preterm birth in the progesterone treatment group, and highest (71.4%) among patients with multiple prior preterm births in the non-treatment group (Figure 4).

Neonatal outcomes are shown in Table 3. The neonatal hospitalization period was shorter in the treatment group (*P* = 0.02), and NICU admissions comprised three newborns (6%) in the treatment group and 14 newborns (25%) in the non-treatment group (odds ratio 0.2; 95% CI 0.05–0.74; *P* = 0.01).

**Table 3:** Neonatal outcomes according to progesterone treatment.

	**Treatment group (*n* = 48)**	**Non-treatment group (*n* = 56)**	***P* value**
Birth weight [g, mean ± SD (range)]	2,834 ± 384 (2,045–3715)	2,849 ± 550 (1,370–4,100)	0.876
Apgar score <7 at 5 min [*n* (%)]	0 (0%)	0 (0%)	–
Neonatal hospitalization [days, mean ± SD, (range)]	4.8 ± 2.5 (2–15)	7.3 ± 6.9 (3–38)	0.017
Neonatal intensive care unit admission [*n* (%)]	3 (6%)	14 (25%)	0.010
Respiratory distress syndrome (RDS) [*n* (%)]	2 (4%)	3 (5%)	1.000
Intraventricular hemorrhage (IVH) [*n* (%)]	0	0	–
Necrotizing enterocolitis (NEC) [*n* (%)]	0	0	–
Retinopathy of prematurity (ROP) [*n* (%)]	0	0	–
Bronchopulmonary dysplasia (BPD) [*n* (%)]	0	0	–
Neonatal death (<28 days) [*n* (%)]	0	0	–

## Discussion

This study evaluated the effect of progesterone treatment in a high-risk population comprised of women with a history of preterm birth and a short cervix diagnosed after 24 weeks of gestation. The main findings are as follows: (1) administration of vaginal progesterone was associated with a lower rate of sPTB before 37 weeks (31% vs. 43%) and before 34 weeks (0% vs. 7%) of gestation as compared to no progesterone treatment; (2) NICU admissions and neonatal hospitalization stays were significantly lower in the progesterone treatment group, and (3) a subgroup analysis evaluating the number of previous preterm births and progesterone treatment demonstrated that multiple prior preterm deliveries significantly increase the recurrence risk of preterm birth, and those not treated with progesterone had a consistently higher rate of preterm birth.

The two main risk factors for preterm birth are previous preterm birth (1, 3–6) and a sonographic short cervix (1, 16, 17, 19, 25, 26). History of spontaneous preterm birth is associated with recurrence of up to 57% in subsequent pregnancies (1, 3–6). Iams et al. reported that a mid-trimester cervical length of ≤ 25 mm increases the relative risk for preterm delivery by 6.19 (19), whereas the preterm prediction study demonstrated that women with prior preterm birth and a mid-trimester short cervix (<25 mm) have a 25% risk of recurrence, ranging up to 65% if a fetal fibronectin test is positive (27). Additionally, vaginal micronized progesterone treatment has been found to significantly reduce preterm birth rates among women with prior preterm birth (7, 9, 10) and women with a mid-trimester short cervix (12–14).

Our study demonstrated that, in a high-risk population cohort, 68.75% of the progesterone treatment group delivered at term, while only 57.2% of the non-treatment group delivered at term. This study may be underpowered to identify the statistically significant difference; however, this finding aligns with previous studies on recurrent preterm birth (1, 3–6, 27) and vaginal progesterone treatment for a short cervix diagnosed in the mid-trimester (12). These findings suggest that, for high-risk patients who present with a late-onset short cervix detected ≥24 weeks of gestation, vaginal progesterone administration may be beneficial.

The study by McManemy et al. evaluated patients with a history of preterm delivery, reporting that those with two previous preterm births had a significantly higher risk for recurrent preterm birth when compared to women with one previous preterm birth (33–57% vs. 13–21%, respectively) (4). Women in the current cohort presented with previous preterm delivery that varied by number and order of appearance. Our findings correlate with the conclusions of McManemy et al. and show that women with multiple previous preterm deliveries presented a higher risk for preterm birth, as demonstrated in Figure 4. The reduction in the preterm birth rate among women treated with vaginal progesterone can be examined in this subgroup analysis: the risk for preterm birth in women with one previous preterm birth ranged from 23.5% (treatment group) to 33.3% (non-treatment group); for women with two or more previous preterm births, the risk ranged from 50.0% to 71.4%. This finding emphasizes the importance of close follow-up in high-risk patients, primarily those with a history of multiple preterm births. Some patients do not receive preventative vaginal progesterone treatment, although recommended. Herein, the study shows that, if pregnant women are diagnosed with a short cervix <25 mm even after 24 weeks of gestation, it is still beneficial to administer vaginal progesterone to prevent preterm birth.

The results of the Cox proportional regression model showed that while the adjusted hazard ratio of the treatment group was not statistically significant, indicating no difference in continued pregnancies between the two groups, the number of previous preterm deliveries was found to be a significant factor. This emphasizes the significance of considering a patient's prior obstetrical history when making treatment decisions regarding progesterone administration.

A comparison of adverse neonatal outcomes reveals a significant decrease in NICU admissions and shorter neonatal hospitalization stays for the newborns of women who received progesterone treatment for diagnosed short cervical length. In fact, the parameters of earlier gestational age at cervical length measurement, tocolytic therapy, antepartum corticosteroid administration, and a cervical length ≤ 15 mm were more prevalent in the treatment group (Tables 1, 2). If the assumption that patients with higher risk were sorted for progesterone treatment were accurate, we would expect a higher rate of sPTB in the treatment group. Yet, this group presented a lower rate of sPTB, suggesting that this population can benefit from vaginal progesterone treatment.

## Strengths and limitations

This study has several strengths. (1) this is the largest cohort reported in the literature for the specific high-risk population (patients with previous preterm birth and a cervical length ≤ 25 mm diagnosed after 24 weeks of gestation); (2) strict exclusion criteria were applied in this cohort, which resulted in a very specific sub-population to evaluate the research question; and (3) inclusion criteria specifically referred to vaginal micronized progesterone 200 mg daily, therefore unified type, route, and dosage of the preventive intervention administration. We excluded women who received other types of progestogens (e.g., hydroxyprogesterone caproate, or 17-OHPC) to focus on a single proposed treatment.

This study also has several limitations. First, its retrospective nature encompasses the possibility of information bias. The intervention group prescribed with vaginal micronized progesterone can present selection bias. For example, women who were diagnosed with a short cervix earlier in pregnancy and had a shorter cervical length were considered at higher risk; and this may have led the attending physician to provide progesterone treatment to the women at higher risk. In fact, parameters such as earlier diagnosis in pregnancy, tocolytic therapy, antepartum corticosteroid administration, and cervical length ≤ 15 mm were more prevalent in the progesterone treatment group (Tables 1, 2). Therefore, if the assumption that patients with higher risk were sorted for progesterone treatment were accurate, we would expect a higher rate of sPTB in the treatment group. On the contrary, women in the progesterone treatment group had better obstetrical and neonatal outcomes, suggesting that this population can benefit from vaginal progesterone treatment. Additionally, the current study comprised of a specific population of high-risk pregnancies with a late short cervix, and the results cannot be generalized to the general population. The results are relevant to other similar populations, but not to the population at large.

### Further investigation

These findings need to be validated by prospective controlled trials. Additionally, further studies can examine patients with various high-risk conditions (e.g., multifetal pregnancy). Future studies can assess the benefit of vaginal micronized progesterone compared to no intervention and evaluate which subpopulation can benefit the most from such treatment.

## Conclusions

The impact of vaginal progesterone in patients with a history of preterm delivery had not been tested on women with a cervical length ≤ 25 mm diagnosed after 24 weeks of gestation (18). To that end, this retrospective cohort study provides relevant information for intervention by vaginal micronized progesterone as opposed to no intervention.

In conclusion, preventive treatment should be selected by evaluating specific characteristics for each population, in which the obstetrical history should be a leading factor (e.g., prior preterm delivery and short cervical length). Patients with more than one prior preterm birth have a higher risk of recurrence, and this risk can be implicated in the management of future pregnancies by targeted preventive care (e.g., high-risk specialist counseling to follow up and consider interventional treatment by vaginal progesterone). The efficacy of vaginal progesterone treatment for decreasing adverse neonatal outcomes suggests that this high-risk population may benefit from cervical assessments even after 24 weeks of gestation in addition to progesterone intervention, if necessary. Further prospective investigation might be necessary to evaluate the efficacy of late second and early third-trimester cervical length measurement.

## Data availability statement

The raw data supporting the conclusions of this article will be made available by the authors, without undue reservation.

## Ethics statement

The studies involving human participants were reviewed and approved by Hadassah Review Board (0446-20-HMO). Written informed consent for participation was not required for this study in accordance with the national legislation and the institutional requirements.

## Author contributions

The study was conceived and designed by DL, SP, RR, DR, and DK, with data acquisition assistance from DL, DR, and SP. The EMR database was built and the data was analyzed by DL, DR, and DK. RH, YS, HH, and YE contributed to the study design and interpretation of the results. The draft manuscript was written by DL, SP, RR, DR, and DK. All authors reviewed and provided critical feedback on the final version. All authors bear responsibility for the accuracy and integrity of the data and analysis and approved the final manuscript for submission.
